# Optimizing Suitable Mechanical Properties for a Biocompatible Beta-Titanium Alloy by Combining Plastic Deformation with Solution Treatment

**DOI:** 10.3390/ma17235828

**Published:** 2024-11-27

**Authors:** Raluca Elena Irimescu, Doina Raducanu, Anna Nocivin, Elisabeta Mirela Cojocaru, Vasile Danut Cojocaru, Nicoleta Zarnescu-Ivan

**Affiliations:** 1 Department of Metallic Materials Processing and Ecometallurgy, University POLITEHNICA of Bucharest, 060042 Bucharest, Romania; raluca.irimescu@stud.sim.upb.ro (R.E.I.); mirela.cojocaru@upb.ro (E.M.C.); dan.cojocaru@upb.ro (V.D.C.); nicoleta.zarnescu@upb.ro (N.Z.-I.); 2 Faculty of Mechanical, Industrial and Maritime Engineering, Ovidius University of Constanța, 900527 Constanța, Romania; anocivin@univ-ovidius.ro

**Keywords:** β-Titanium alloys, XRD, SEM, tensile tests, mechanical properties

## Abstract

The microstructural and mechanical features were investigated for the alloy Ti-36.5Nb-4.5Zr-3Ta-0.16O (wt.%) subjected to thermo-mechanical processing consisting of a series of hot and cold rolling combined with solution treatments with particular parameters. The objective was to find the optimal thermo-mechanical treatment variant to improve the mechanical properties, and namely, to increase the yield tensile strength (YTS) and the ultimate tensile strength (UTS), with a low modulus of elasticity and with an adequate ductility in order to obtain a good biomaterial appropriate for use in hard tissue implants. X-ray diffraction and SEM microscopy served to investigate the microstructural features: the type of formed phases with their morphology, dimensions, and distribution. The experimental alloy presented mainly a β-phase with some α″-Ti martensitic phase in particular stages of the processing scheme. The main mechanical properties were found by applying a tensile test, from which were determined the yield tensile strength [MPa], the ultimate tensile strength [MPa], Young’s modulus of elasticity [GPa], and the elongation to fracture (%).

## 1. Introduction

Of the titanium alloys used as biomaterials for load-bearing implant manufacture, the metastable β-Titanium alloys are considered the most promising ones due to their easier processability due to their bcc type of microstructure [1,2,3,4] and low elastic modulus of around 48–70 GPa [5,6,7], which can prevent the unwanted “stress shielding” phenomenon. However, compared to two other possible Ti-alloys, α- or α/β-type, the β-Ti alloy has a moderate yield strength (of around 350–400 MPa [8,9,10,11]), thus not complying with the imposed mechanical requirements for medical applications such as permanent implants. For this kind of application, β-Ti alloys are exposed to varying stress levels during daily activities, from extreme stress during physical activity, to low stress during periods of rest [10,12]. As a result, high strength and resistance to multiaxial fatigue loading combined with good ductility are necessary for an adequate β-Ti alloy [13,14].

Thus, in all reports in the literature, the system alloy TNTZ (Ti-Nb-Ta-Zr) has the most promising characteristics for orthopedic applications [15,16], due to, first of all, the high biocompatibility of all the alloying elements, that, in addition, exhibit a high corrosion resistance in the environment of the human body as well. Niobium and tantalum are well-known β-stabilizer elements, niobium being the strongest [17,18,19]. Therefore, the niobium content in these alloys should be quite high, at around 30–40 wt.%. This way, the β-type microstructure can be considered to be secure. Zirconium, for its part, is generally considered a neutral element, with negligible effect on the stability of both the α- and β-phases [20]. However, many reports have established that zirconium, even though it is considered a neutral element, can favor the stabilization of the β-phase in the presence of β-stabilizers [20,21,22] such as Nb and Ta. In addition, the toughness of Ti alloys can be increased by zirconium, as with niobium, if it is incorporated into the Ti alloy composition [21,22,23], and it is also associated with a supplementary decrease in the elastic modulus [23]; another important and beneficial aspect is that the presence of zirconium can ensure the suppression of the formation of the omega phase [23], which is the phase that seriously endangers the mechanical characteristics of these alloys.

As a result, although they are strongly biocompatible according to their chemical composition, TNTZ alloys with a single β-phase have a 0.2% yield stress (0.2%YS) of about 400–550 MPa [24], while that of Ti-6Al-4V alloy is about 800–880 MPa [25]. Consequently, the challenge for these β-type TNTZ alloys is to increase the yield strength while maintaining an acceptable level of ductility and as low a Young’s modulus as possible. Generally, there are two modalities of action: (1) to optimize the chemical composition and (2) to design a suitable thermo-mechanical processing method for the alloy.

Referring to the first modality, the addition of oxygen to TNTZ-type alloy represents one of the more recently proposed innovative directions. Oxygen is a strong interstitial strengthening element [26,27] of the β-phase, consequently acting as a β-stabilizer, being able to inhibit stress-induced martensitic transformation (β→α″) [28,29,30], even though, at the same time, it is considered an α-stabilizing element because it causes an increase in the β-transus temperature of the titanium alloy [31]. Its strengthening effect is reported to be more influential than its α-stabilizing characteristic [31,32,33].

From all the recently published reports [34,35,36,37,38], the conclusion is that it is difficult to evaluate the influence of only oxygen on the alloy’s properties without taking into account the influence of the other β-stabilizing alloying elements, combined with the influence of thermo-mechanical treatments that can be applied simultaneously.

Therefore, the second modality of increasing the strength—the thermo-mechanical processing of the alloy—is considered the most important and influential modality of action. It consists of a combination of plastic deformations, which ensures great refinement of the granulation with an obvious increase in the yield point with the solution treatments (STs), which in turn ensure adequate ductility without greatly affecting the high level of strength already obtained.

The most recent works regarding the mechanical property evolution as a result of both treatment routes, thermo-mechanical treatment and oxygen addition, report the following data: the Ti-35Nb-6Ta-7Zr-2Fe-0.4O alloy, in [28], achieved a UTS of 1130 MPa with a Young’s modulus (E) of around 70 GPa; the Ti-25Nb-17Ta-1Fe-0.25O alloy, in [34], achieved a UTS of 851 MPa, with an E of about 60 GPa; the Ti-34.18Nb-7.6Zr-0.9Fe-0.16O alloy, in [35], achieved a UTS of 1142 MPa, with a Young’s modulus (E) of around 49 GPa; the Ti-29Nb-13Ta-4.6Zr-xO alloy, with two different oxygen contents (2200 and 4600 ppm), in [36], achieved a microhardness of about 400 HV, with an E between 53 and 66 GPa; the Ti-7.5Mo-xO (x ≤ 0.3) alloys, in [37], achieved a combination of a YTS of ~640 MPa, an elongation of ~28%, and an E of ~60 GPa; the Ti-35%Ta-1,5O (at%) alloy, in [38], achieved a 0.2%YS of 788 MPa.

As can be seen, all of these reports refer to a fairly wide range of values, implying that many other experimental programs can still be carried out with experimental parameters that can be optimized, both in terms of the chemical composition and those referring to the processing variants of the alloys involved.

Accordingly, for the present study, a β-Ti alloy has been selected, of the Ti-Nb-Zr-Ta type with oxygen, that has not been tested before, in order to achieve suitable mechanical properties (a low Young’s modulus with high strength) through the simultaneous implementation of both intervention routes: (1) oxygen addition; (2) thermo-mechanical processing of the alloy with a series of plastic deformations coupled with super-transus and near-transus solution treatments with optimized parameters. Hence, the main objective is to find a proper processing route for a β-Ti alloy with oxygen, suitable to be proposed as implant material.

## 2. Materials and Methods

### 2.1. Synthesis of the Selected for Study Alloy

The chemical composition considered for the titanium alloy proposed for study was Ti-36.5Nb-4.5Zr-3Ta-0.16O (wt.%). For the synthesis of the alloy, a levitation induction melting furnace FIVE CELES-MP25 (Five’s Group Company, Paris, France) with a high vacuum of 10^−4^–10^−5^ mbar and with intense agitation of the melted composition was used. High-purity titanium (min. 99.7%), niobium (min. 99.9%), zirconium (min. 99.6%) and tantalum (min. 99.9%) were used to obtain the necessary ingots of the alloy. The small amount of oxygen (O) results from the composition of other elements, mainly titanium. To ensure a high degree of chemical homogeneity, the ingots were re-melted several times.

The rationality for selecting the above chemical composition consisted in using the following elements: niobium (36.5 wt.%), first of all, as the strongest β-stabilizing alloying element; zirconium and tantalum, also, as highly biocompatible elements, but to a lesser extent (4.5 wt.% and 3 wt.%, respectively); and an amount of 0.16 wt.% oxygen, in order to obtain an increase in the alloy strength.

The dimensions of the initial ingot were ∅ 20 × 45 mm, from which samples with the dimensions of 2 × 15 × 45 mm were prepared by cutting for the experiments provided below, using a Metkon MICRACUT 200 type machine (Metkon Instruments Inc., Bursa, Turkey).

### 2.2. The Designing of the Thermo-Mechanical Processing Schema of the Alloy

**Figure 1:**
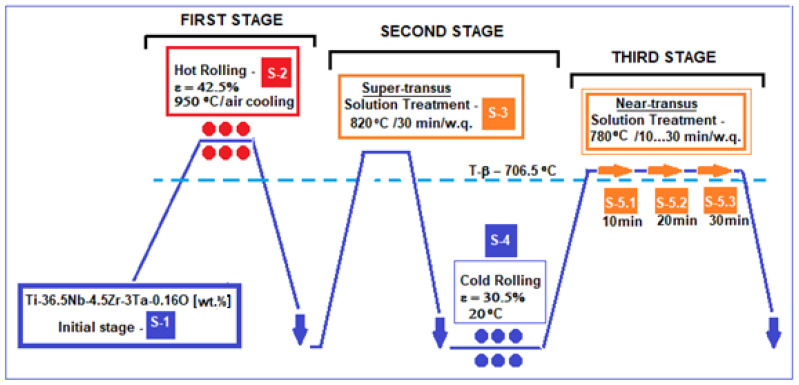
Schema of thermo-mechanical processing of the Ti-36.5Nb-4.5Zr-3Ta-0.16O (wt.%) alloy.

The thermo-mechanical processing scheme used for the present experimental program is shown in Figure 1.

In order to design the experimental program for the thermo-mechanical processing of the alloy, the first aspect considered was the determination of the α → β transition temperature, named the β-transus temperature, depending on which the heating temperatures for the intended thermo-mechanical treatments can be established.

Thus, the empirical formula was used (Equation (1) below), determined by Sun et al. [39], which is more comprehensive than those previously stated [40], because it refers to a wider set of 19 chemical elements, including the alloying elements used in the studied alloy—niobium, tantalum, zirconium, and oxygen:

T(α/α + β) = 882 °C + ∑ Fi (Xi)(1)

The polynomial linear regression method is used for this formula to show the effect of each chemical element T(βi) = Fi (Xi) on the β-transus temperature in a form of a second- or third-order polynomial. Table 1 presents the resulting expression for each alloying element used for the present case. Thus, applying Equation (1) for the studied alloy Ti-36.5Nb-4.5Zr-3Ta-0.16O (wt.%), or Ti-24.04Nb-3.02Zr-1.01Ta-0.61O (%at.), a β-transus temperature equal to 706.5 °C resulted.

As a result, below are presented the intended stages of the designed experimental program, according to Figure 1:

- S-1 initial state, corresponding to the initially obtained alloy ingot, is considered to be the starting microstructure for the experimental program.
- S-2 state, which corresponding to that obtained from hot rolling (HR) at 950 °C, with a relative reduction in ε = 42.5%, followed by air cooling, and using a Mario di Maio LQR120AS rolling-mill (Mario di Maio Inc., Milan, Italy); the rolling speed was about 3 m/min. This first stage of the thermo-mechanical treatment scheme provides the highest heating temperature (950 °C) to ensure the simultaneous development of both microstructural processes: granulation reduction through plastic deformation, and recrystallization through high temperatures.
- S-3 state, corresponding to solution treatment above the β-transus temperature, therefore named a super-transus solution treatment: at 820 °C, with a holding time of 30 min (enough by calculation to solubilize the 2 × 15 × 45 mm sample), followed by water quenching (w.q.) in order to preserve the β-bcc microstructure obtained through this heat treatment. A GERO SR 100 × 500-type oven (Carbolite-Gero Inc., Neuhausen, Germany) under a high vacuum was used.
- S-4 state, corresponding to cold rolling (CR) with the relative reduction in ε = 30.5% using the same Mario di Maio LQR120AS rolling-mill as for HR. No lubricant was used. For this operation, an ultrasonic bath at 60 °C in ethylic alcohol was used for cleaning the samples.
- Obtaining samples S3 and S4 corresponded to the second stage of the experimental program, also aiming for a high reduction in the grain size like for the first stage, this time with texturing of the microstructure at the end, after the applied CR. This step involved first obtaining a homogeneous β-type microstructure (S-3), which then allows for easier cold rolling processing (S-4).
- S-5 state, corresponding to solution treatment very close to β-transus temperature, named near-transus solution treatment, at 780 °C, with three variants of holding time: S-5.1, 10 min; S-5.2, 20 min; S-5.3, 30 min. It follows water quenching (w.q.) to preserve the obtained microstructure. For this stage, the same equipment as for S-3 was used. The purpose of this third stage was to try three different holding times to see which one can achieve a more suitable microstructure with optimized mechanical properties at a temperature much closer to β-transus (780 °C) to save energy.

### 2.3. Analysis of the Alloy Microstructure and of Mechanical Properties

The samples resulting from all the experimental stages were subjected to microstructural and mechanical analysis as well. The microstructural analysis sought to highlight the evolution of the particularities of the microstructure, related to the type of phases, their characteristic morphology and dimensions, in accordance with the stages of thermo-mechanical processing. To achieve this goal, two analyses were performed at room temperature: one by X-ray diffraction (XRD) and one by scanning electron microscopy using the electron backscatter diffraction (EBSD) technique.

For XRD analysis, a Philips PW 3710 diffractometer was used, with radiation source of Cu k-alpha, using a scan range between 30° and 90° for 2θ (°) with a step size of 0.02°. The projected direction of the X-rays was set to be parallel to the rolling direction for both the hot rolling and cold rolling procedures. The MAUD v2.33 software package was used for the XRD spectra simulation; thus, the phase lattice parameters were determined. On the other hand, the PeakFit v4.12 software package was used for the fitting procedure of the recorded XRD spectra; the position and intensity of each peak, and the peak broadening—FWHM (Full Width at Half Maximum)—were determined.

For EBSD-SEM analysis, a TESCAN VEGA II—XMU microscope (Tescan Orsay Holding, a.s., Brno, Czech Republic) was used, provided with a BRUKER eFlash1000 EBSD detector (Bruker Corporation, Billerica, MA, USA). The parameters of the EBSD measurements were as follows: 512 × 512 pixel image size, 320 × 240 pixel EBSD resolution, 10 ms acquisition time/pixel, 1 × 1 binning size, and less than 2% zero solutions. The samples that were plastically deformed, at either hot or cold temperatures (S-2 and S-4), were examined in the cross-section of the RD–ND (RD—rolling direction; ND—normal direction). The metallographic samples were prepared by using the following equipment: for the cutting procedure, a Metkon MICRACUT 200-type machine (Metkon Instruments Inc., Bursa, Turkey) with diamond cutting disks; for fixing the samples, a Metkon Digiprep ACCURA machine (Metkon Instruments Inc., Bursa, Turkey), using a specific epoxy resin of a Buehler Sampl-Kwick type, abraded with 1200 grit SiC paper; for mechanical polishing of the metallographic samples, a Buehler VibroMet2 machine (Buehler Ltd., Lake Bluff, IL, USA), using polycrystalline diamond suspension of 6, 3, and 1 μm successive fineness, followed then by 0.03 μm colloidal silica.

A Gatan MicroTest-2000N-type machine (Gatan Inc., Pleasanton, CA, USA) with a strain rate of 1 × 10^−4^ s^−1^ was used to perform tensile tests for all experimental stages on tensile test samples with a dog bone form with a calibrated area of 0.8 × 6 × 20 mm. The strain–stress curves resulted, with the help of which the following mechanical characteristics were determined with corresponding standard deviation (SD): the yield strength—σ_0.2_, [MPa]; the ultimate tensile strength—σ_UTS_, [MPa]; the elongation to fracture—ε_f_, [%]; and the elastic modulus—E, [GPa].

## 3. Results

### 3.1. Analysis of the Alloy Microstructure Evolution

Beta-titanium alloys, due to their bcc structure, are considered remarkably workable, both when cold and annealed at room temperature. The alloy proposed for study is a β-metastable alloy if we consider the high amount of β-stabilizing alloying elements, especially niobium (36.5 wt.%).

The reason for applying the thermo-mechanical process in Figure 1 is to obtain a structure formed entirely of β-phase, but with improved mechanical properties that allow the alloy to be used as a suitable biomaterial for orthopedic implants. Therefore, the experimental program designed consists of a combination of hot and cold rolling with solution treatments to obtain a single β structure, with dimensionally fine and homogeneous grain size, with the highest possible strength.

Considering that some titanium alloys, with a similar amount of niobium to that used in the present case, have been successfully solution-treated at 950 °C [41,42], hot rolling applied on the as-cast ingot (S-1) was considered at 950 °C also, having not only chemical homogenization but also grain refining of the structure through applied rolling. Further, the proposed solution treatments were set at 820 °C (for the second stage) and 780 °C (for the third stage), respectively.

According to [41,43], solution treatment is called “super-transus” if the heating temperature is around 850–900 °C. Therefore, for the present case, the name super-transus was also used for the solution treatment applied at 820 °C, while the name “near-transus” was chosen for the solution treatment applied at 780 °C, due to a much closer temperature to β-transus. Cold rolling was interposed between the two solution treatments to achieve a more pronounced reduction in the β granulation.

For the microstructural analysis, it was considered useful to determine the Mo(eq) parameter to evaluate a certain β-stabilizing level of the structure. Thus, according to [41,44], the following Equation (2) has been used, considering only the contents of niobium and tantalum for the present case:

Mo(eq) = 1.0 × (wt.% Mo) + 0.67 × (wt.% V) + 0.44 × (wt.% W) + 0.28 × (wt.% Nb) + 0.22 × (wt.% Ta) + 2.9 × (wt.% Fe) + 1.6 × (wt.% Cr)—1.0 × (wt.% Al)(2)

The resulting Mo(eq) is 10.88%. According to [41], if a titanium alloy has a Mo(eq) between 10 and 30%, it is considered metastable, heat-treatable, and deeply hardenable. For the present case, it turns out that the calculated value of Mo(eq) is very close to the lower limit, a characteristic that will be helpful in the following microstructural analysis.

**Figure 2:**
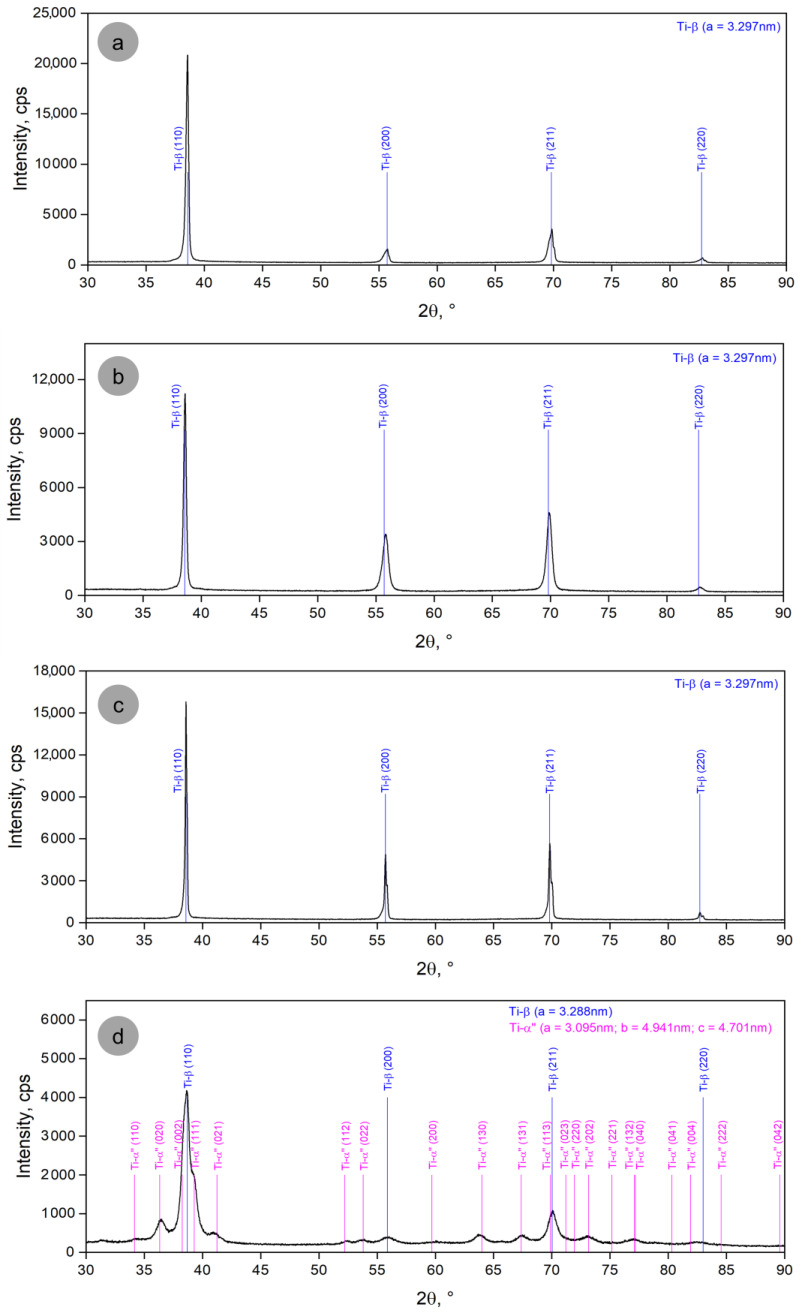
XRD spectra corresponding to first and second stage of the experimental program: (**a**) S-1, the initial as-cast state; (**b**) S-2, HR (950 °C/30 min/air c.); (**c**) S-3, ST (820 °C/30 min/w.q.); (**d**) S-4, CR (20 °C/ε = 30.5%).

Using XRD and EBSD-SEM analysis, all of the experimental stages of the studied alloy were investigated. Beginning with the first two stages of the experiments, Figure 2 presents the XRD analysis with the corresponding results. One can observe that the S-1, S-2, and S-3 states (Figure 2a–c), but not S-4 (cold-rolled; Figure 2d), show only four intense diffraction peaks, (110)_β_, (200)_β_, (211)_β_, and (220)_β_, corresponding to the β-phase, with a bcc crystallographic structure and space group Im3m, labeled according to ICDD no. 04-002-8708 [45]. The determined lattice parameter for the single β-phase is a_β_ = 3.297 Å (0.3297 nm). The presence of only a single β-phase demonstrates the β-stabilizing character of the alloying elements used. Considering that the ordinary lattice parameter for the Ti-β phase is around a_β_ = 3.282 Å (COD 9,012,924 file), one can observe an increase in this parameter for the present formed β-phase, attesting that the dissolved alloying elements within the initial Ti-β phase cause an increase in the lattice parameter to a_β_ = 3.297 Å. The substitutional solid solution, the formation of which is supported by the empirical Begard’s law [46,47], should be considered since all the alloying elements used (niobium, tantalum, and zirconium) have a large atomic radius.

However, for the cold-rolled sample, the appearance of diffraction peaks corresponding to the α″-martensitic phase (orthorhombic system—space group Cmcm) can also be observed, indicating that the Mo(eq) value of 10.88%, located at the lower limit of the metastable range (10–30%), implies/certifies the possibility of the precipitation of the martensitic phase as a result of the stress-induced martensitic transformation (SIM) caused by cold rolling. Indeed, if one calculates the starting temperature of the martensitic transformation—Ms, using data from [48], a value of 55.2 °C is obtained, which confirms the possibility that at room temperature, through cold rolling, the SIM transformation can be induced. As a result, the appearance of diffraction peaks corresponding to the α″-phase (Figure 2d) is perfectly plausible. The determined lattice parameters for orthorhombic α″-phase were as follows: a_α_ = 3.095 Å; b_α_ = 4.941 Å; c_α_ = 4.701 Å.

**Figure 3:**
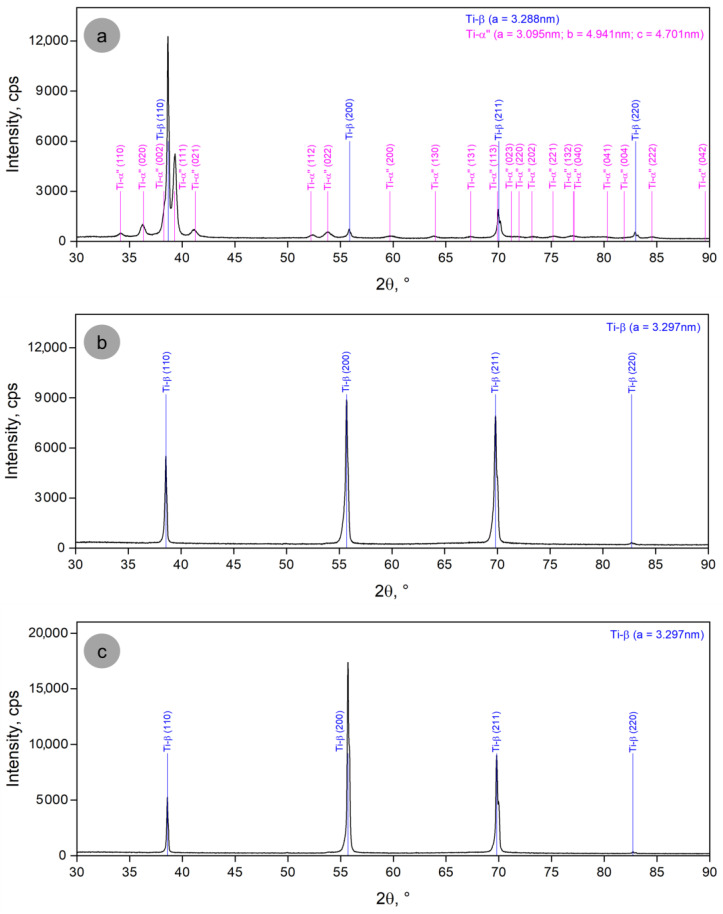
XRD spectra corresponding to third stage of the experimental program: (**a**) S-5.1, ST/10 min; (**b**) S-5.2, ST/20 min; (**c**) S-5.3, ST/30 min.

Figure 3 presents the XRD spectra of the sample states from the third stage of the experiments: S-5.1, S-5.2 and S-5.3, respectively. The XRD spectra from Figure 3a show peaks corresponding to both the β-metastable phase and the α″-martensitic phase, a fact attributed to the very short holding time at the temperature of 780 °C (near-transus), during which the complete dissolution of the martensite in the solid solution cannot be achieved. However, the following two images (Figure 3b,c), corresponding to longer holding times—20 min and 30 min—indicate only the peaks specific to the β-phase, meaning that the martensite had enough time to completely dissolve in the solid solution during the near-transus treatment.

**Figure 4:**
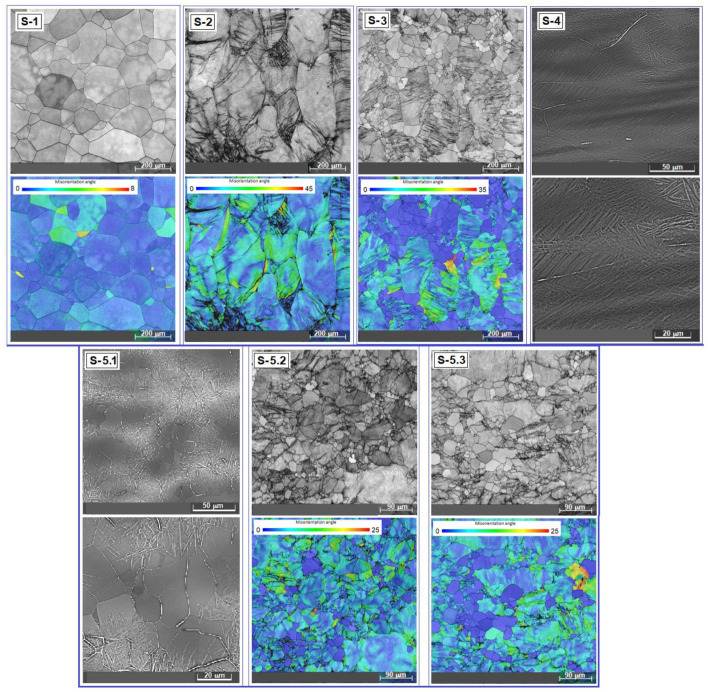
SEM images of the studied alloy corresponding to all stages of the experimental program: S-1, as-cast initial state: SEM-EBSD image and MO map; S-2, HR state: SEM-EBSD image and MO map; S-3, solution treatment: SEM-EBSD image and MO map; S-4, CR: two SEM-BSE images; S-5.1, ST/10 min: two SEM-BSE images; S-5.2, ST/20 min: SEM-EBSD image and MO map; S-5.3, ST/30 min: SEM-EBSD image and MO map.

Figure 4 presents SEM-EBSD images for all of the tested stages of the studied alloy. The first two images correspond to S-1, the as-cast initial state (one is the SEM-EBSD image and the other is the MO modal orientation distribution map). One can observe that the microstructure shows the equiaxed polygonal homogeneous grains of the β-phase. The area of the strained grains is reduced, meaning that the repeated re-melting procedure during the synthesis process has a beneficial influence.

Following the two series of images from Figure 4, corresponding to the S-2 (HR) and S-3 states (super-transus ST), each with an SEM-EBSD image coupled with a distribution map of the MO modal orientation, a larger quantity of dimensionally heterogeneous and strained grains is indicated, which is more accentuated in S-2 than in S-3, with visible local deformations (hatched areas). Compared to S-2 (HR), the S-3 state (super-transus ST) has smaller grains with a strained character still present for some of them. The areas with small and homogeneous grains in S-3 represent the new recrystallized ones, formed from the previous hot rolled stage and with a strongly deformed character (the hatched areas). As a result, S-3 shows the simultaneous existence of two visibly distinct areas: some with small and homogeneous grains (recrystallized ones) and others with larger grains, but smaller than those in S-2, and with the persistent appearance of deformation and a strained character.

The series of two SEM-BSE images in Figure 4 corresponding to S-4 (CR state at two different magnifications) shows a highly deformed microstructure, consisting of α″ martensite with an acicular morphology, which is formed due to the SIM transformation, mixed with the β-phase. Correlated with Ms = 55.2 °C, this microstructure appearance is in concordance with the XRD spectra previously discussed (Figure 2d).

The SEM-BSE images corresponding to the S-5.1 state—ST/10 min (two different magnifications) show undeformed equiaxial grains with a slight dimensional non-uniformity, with the presence of an overlapping acicular structure for some grains, a sign that the martensitic α″ phase is still present alongside the β-phase. This microstructural feature is in agreement with the XRD spectra in Figure 3a, attesting that holding for only 10 min at 780 °C is not sufficient for the martensitic phase to completely dissolve into the β-phase.

The last two series of the images from Figure 4, corresponding to the S-5.2 and S-5.3 states (an SEM-EBSD image with the corresponding modal orientation (MO) distribution map), show the most dimensionally uniform and small granulation out of all of the treated samples, with a sufficiently strained state that justifies the obtained strengthening character discussed below. It can be appreciated that there are no visible morphological differences between the two states. It will also be seen that the mechanical properties of these two states are relatively close in value.

As a conclusion for the microscopic analysis carried out for all the tested samples, it can be appreciated that following the combined plastic deformation and solution treatment program, one of the initially proposed objectives, that of refining the granulation of the studied alloy, was fulfilled by the fact that there is a visible decrease in the grain dimension between the initial granulation (S-1) and the final one (S-5.2 and S-5.3), of about 1/3, which represents a satisfactory result.

### 3.2. Analysis of the Alloy Mechanical Property Evolution

The microstructural analysis is completed by the results related to the mechanical properties from the tensile test also performed on all of the states of the processed alloy.

**Figure 5:**
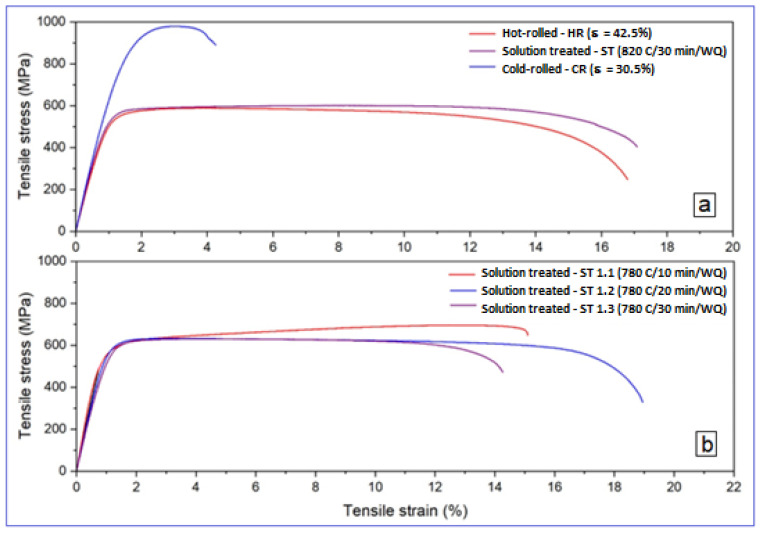
The strain–stress curves corresponding to all stages of the studied alloy: (**a**) the strain–stress curves for the first and second stages of the experiments; (**b**) the strain–stress curves for the third stage of the experiments.

**Figure 6:**
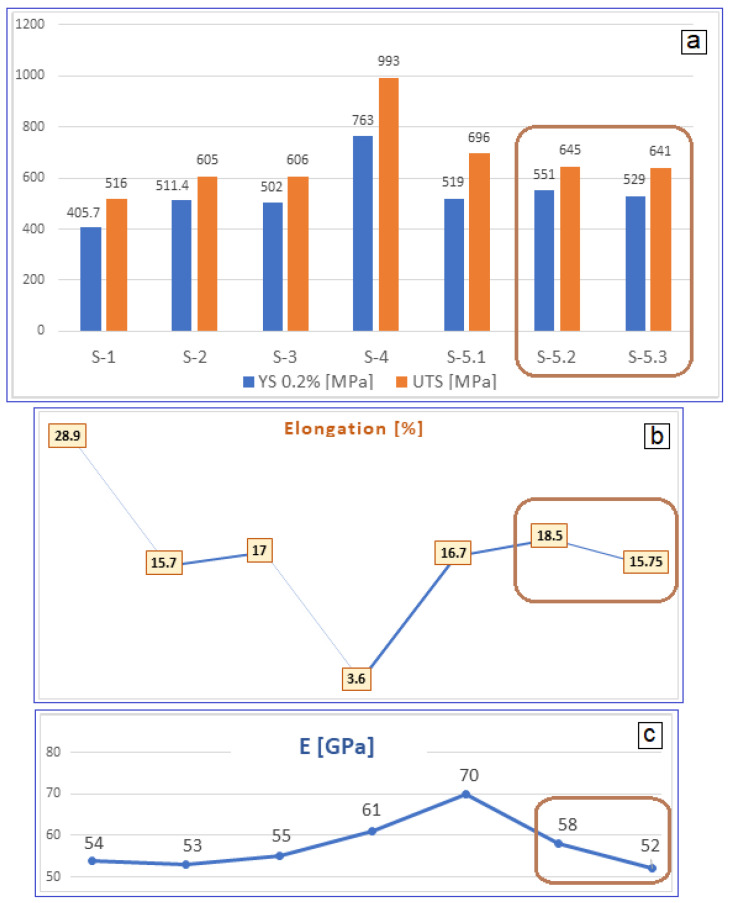
Mechanical property evolution of the studied alloy for all stages of the experimental program: (**a**) ultimate tensile strength [MPa]—UTS; yield strength [MPa]—(YS); (**b**) elongation to fracture (%)—ε; (**c**) Young’s modulus [GPa]—E.

Figure 5 shows the strain–stress curves obtained after the applied tensile tests. Table 2 indicates for all the tested samples the values of the mechanical properties determined from the strain–stress curves. Figure 6 indicates the evolution of all the mechanical properties determined.

Table 2, in conjunction with Figure 6, indicates an increasing evolution of YS and UTS, starting from the homogenized state S-1, up to S-4 (CR), for which the highest values are recorded, after which the values start to decrease. Thus, for the following three states (S-5.1 (ST-10 min), S-5.2 (ST-20 min) and S-5.3 (ST-30 min)), the YS and UTS values decrease a little; the last two states, corresponding to S-5.2 and S-5.3, present relatively uniform mechanical values, with very close UTS values of about 645 and 641 MPa, respectively. For these last two states, Young’s modulus of 52 and 58 GPa, combined with the elongation to fracture of 18.5% and 15.75%, respectively, represent encouraging values, with a promising combination of mechanical properties: good reinforcement, a low elastic modulus, and a sufficiently large elongation. This can be considered the second relevant result obtained through this experimental program.

The Young’s modulus of the Ti-alloys is unanimously considered as being directly related to the e/a ratio (valence electrons per atom) [10,49]. Thus, if the alloying elements with a β-stabilizing character are present at a high amount (in the present case, at about 40wt.%), the e/a also has a higher value, representing the strong stabilization of the β-phase [50,51].

As a result, it is reported that if the values of the e/a ratio are between 4.24 and 4.26, the resulting modulus can achieve much smaller values for the β-Ti alloys [49,50,51,52]. The e/a ratio calculated for the present case is 4.2324, a value close to the 4.24–4.26 interval. Therefore, the obtained promising values for the modulus of about 52–58 GPa could confirm this theory. Moreover, these values are compared with those of other β-Ti-type alloys with close chemical composition and e/a values belonging to the interval (4.24–4.26):

(a) UTS = 500 MPa; E = 63 GPa for the Ti-35.3Nb-7.1Zr-5.1Ta wt.% [53];
(b) UTS = 500 MPa; E = 65 GPa for the Ti-41.1Nb-7.1Zr wt.% [4];
(c) UTS = 641 MPa; E = 52 GPa for the Ti-36.5Nb-4.5Zr-3Ta-0.16O wt.% [present case];
(d) UTS = 755 MPa; for the Ti-39Nb-6Zr-0.26O wt.% [19];
(e) UTS = 851 MPa; E = 60 GPa for the Ti-25Nb-17Ta-1Fe-0.25O wt.% [5].

By comparing, one can appreciate that the obtained values can be considered to be promising. In addition, for the compared alloys, an increase in the UTS value can be observed with the addition of oxygen to the composition.

Another important aspect of the presented results is related to the addition of oxygen, whose strong interstitial strengthening influence is reported in many studies [54,55,56,57]. Observing the comparison made above with some alloys with similar compositions and processing methods, it can be stated that the presence of oxygen can indeed increase the strength of the β-matrix: the oxygen-free alloys in examples (a) and (b) have lower UTS and higher E values compared to those with low oxygen addition. Even the present alloy falls within this range according to the obtained values of the mechanical characteristics.

## 4. Conclusions

A complex experimental program of thermo-mechanical processing, involving a series of hot and cold rolling treatments alternated with super-transus and near-transus solution treatments, was designed for a β-Ti alloy, that has been previously untested: Ti-36.5Nb-4.5Zr-3Ta-0.16O (wt.%). This processing program aimed to find an optimal variant with adequate mechanical characteristics that could be proposed for use in the medical field.

For the final near-transus solution treatment applied, three different holding times were applied: 10 min, 20 min, and 30 min.

The microstructural analysis, performed with X-ray diffraction and SEM microscopy, revealed a refinement of the majority β-phase granulation by approximately one-third compared to that originally obtained in the as-cast sample.

The mechanical characteristics determined by the tensile tests showed that, at the end of the processing program, sufficiently higher and uniform values of YS and UTS (around 551 and 645 MPa, respectively) were obtained, associated with improved elongation values (around 17%). The final Young’s modulus had low values of approximately 52–58 GPa, which represented an encouraging result.

Through comparing the mechanical characteristics obtained for the studied alloy (i.e., UTS and E) with those of alloys with similar compositions and made through similar processes previously reported in the specialized literature, it was concluded that the results obtained can be considered promising.

## Figures and Tables

**Table 1:** Formula T(βi) = Fi (Xi) for the used alloying elements of the studied alloy, conforming to [39].

Alloying Element	T(βi) [°C]	Applicable Concentration Range [%]
Niobium	−12.1312 × [Nb] + 0.08178 × [Nb]^2^ − 0.000334771 × [Nb]^3^	0–40
Tantalum	−7.4877 × [Ta] + 0.13494 × [Ta]^2^ − 0.00175 × [Ta]^3^	0–30
Zirconium	−3.53793 × [Zr] − 0.04004 × [Zr]^2^ − 0.00037309 × [Zr]^3^	0–40
Oxygen	134.88076 × [O] + 21.00293 × [O]^2^ + 8.39629 × [O]^3^	0–1

**Table 2:** Mechanical properties determined for the experimental samples of the studied alloy: ultimate tensile strength [MPa]—UTS; yield strength [MPa]—(YS); elongation to fracture (%)—ε; Young’s modulus [GPa]—E.

Sample		UTS [MPa]	YS [MPa]	E [GPa]	ε [%]
S-1	Test 1	537.16	412.54	55.17	35.48
	Test 2	495.40	398.94	53.51	22.46
	Average	516.28 ± 20.88	405.74 ± 6.80	54.34 ± 0.83	28.97 ± 6.51
S-2	Test 1	588.09	498.31	53.30	16.79
	Test 2	622.26	524.50	54.23	14.61
	Average	605.18 ± 17.09	511.41 ± 13.10	53.77 ± 0.47	15.70 ± 1.09
S-3	Test 1	600.51	499.86	56.39	17.08
	Test 2	611.19	504.13	53.62	16.99
	Average	605.85 ± 5.34	502.00 ± 2.13	55.01 ± 1.39	17.04 ± 0.04
S-4	Test 1	978.65	731.13	59.21	4.25
	Test 2	1.009.20	796.57	63.84	3.03
	Average	993.93 ± 15.28	763.85 ± 32.72	61.53 ± 2.32	3.64 ± 0.61
S-5.1	Test 1	694.72	517.46	69.81	15.10
	Test 2	697.27	521.56	70.35	18.32
	Average	696.00 ± 1.27	519.51 ± 2.05	70.08 ± 0.27	16.71 ± 1.61
S-5.2	Test 1	632.02	541.97	57.41	18.94
	Test 2	657.34	561.17	58.65	18.04
	Average	644.68 ± 12.66	551.57 ± 9.60	58.03 ± 0.62	18.49 ± 0.45
S-5.3	Test 1	629.32	525.56	52.08	14.25
	Test 2	652.50	532.34	53.39	17.25
	Average	640.91 ± 11.59	528.95 ± 3.39	52.74 ± 0.66	15.75 ± 1.50

## Data Availability

The data are contained within the article.
